# Phosphorylation of protein kinase A (PKA) regulatory subunit RIα by protein kinase G (PKG) primes PKA for catalytic activity in cells

**DOI:** 10.1074/jbc.M117.809988

**Published:** 2018-01-29

**Authors:** Kristofer J. Haushalter, Darren E. Casteel, Andrea Raffeiner, Eduard Stefan, Hemal H. Patel, Susan S. Taylor

**Affiliations:** From the Departments of ‡ Chemistry & Biochemistry,; ¶ Medicine,; § Anesthesiology, and; ‡‡ Pharmacology, University of California, San Diego, La Jolla, California 92093-0654,; the ‖ Institute of Biochemistry, University of Innsbruck, A-6020 Innsbruck, Austria, and; the ** Veterans Affairs San Diego Healthcare System, San Diego, California 92161

**Keywords:** phosphorylation, post-translational modification (PTM), protein kinase, protein kinase A (PKA), protein kinase G (PKG), PKA Regulatory Subunit RI alpha (RI&α)

## Abstract

cAMP-dependent protein kinase (PKAc) is a pivotal signaling protein in eukaryotic cells. PKAc has two well-characterized regulatory subunit proteins, RI and RII (each having α and β isoforms), which keep the PKAc catalytic subunit in a catalytically inactive state until activation by cAMP. Previous reports showed that the RIα regulatory subunit is phosphorylated by cGMP-dependent protein kinase (PKG) *in vitro*, whereupon phosphorylated RIα no longer inhibits PKAc at normal (1:1) stoichiometric ratios. However, the significance of this phosphorylation as a mechanism for activating type I PKA holoenzymes has not been fully explored, especially in cellular systems. In this study, we further examined the potential of RIα phosphorylation to regulate physiologically relevant “desensitization” of PKAc activity. First, the serine 101 site of RIα was validated as a target of PKGIα phosphorylation both *in vitro* and in cells. Analysis of a phosphomimetic substitution in RIα (S101E) showed that modification of this site increases PKAc activity *in vitro* and in cells, even without cAMP stimulation. Numerous techniques were used to show that although Ser^101^ variants of RIα can bind PKAc, the modified linker region of the S101E mutant has a significantly reduced affinity for the PKAc active site. These findings suggest that RIα phosphorylation may be a novel mechanism to circumvent the requirement of cAMP stimulus to activate type I PKA in cells. We have thus proposed a model to explain how PKG phosphorylation of RIα creates a “sensitized intermediate” state that is in effect primed to trigger PKAc activity.

## Introduction

**Figure 1.**
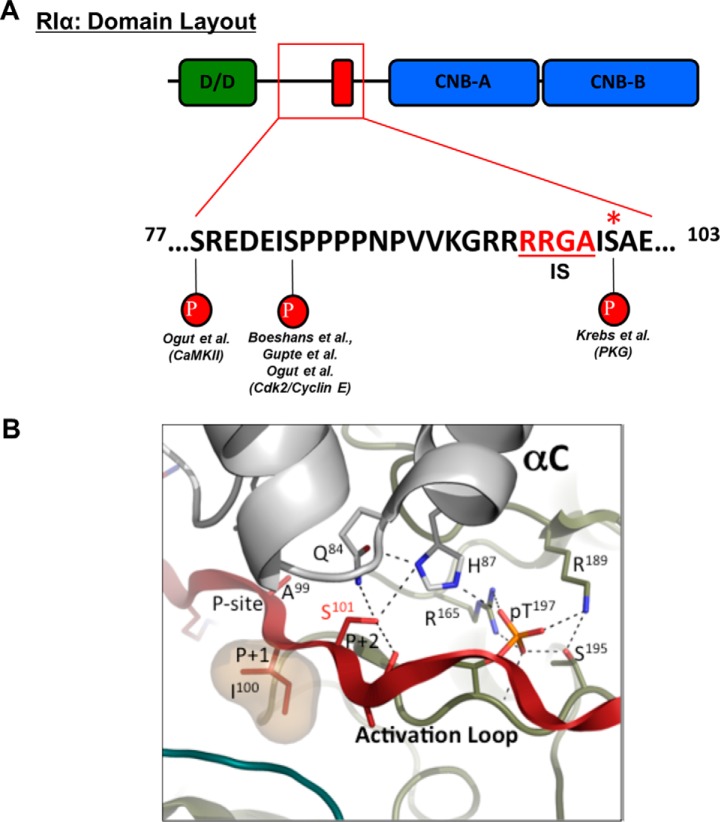
*A*, cartoon diagram of RIα domain layout, with emphasis on the linker region sequence and phosphorylation sites Ser^77^, Ser^83^, and Ser^101^. RIα domains from the N terminus to the C terminus were as follows: dimerization/docking (*D/D*) domain, the pseudosubstrate inhibitory site (IS; sequence is shown in *red underlined text*), and tandem CNB domains. The presumed kinases for each putative phosphorylation site are listed below. The PKG phosphorylation site Ser^101^ is indicated by a *red asterisk. B*, PyMOL structural representation of the RIα–PKAc heterodimer complex (Protein Data Bank code 2qcs), focusing on the binding interface between the active site of PKAc and the linker region IS of RIα (*light-gray cartoon*, PKAc N-lobe; *olive-green surface*, PKAc C-lobe; *teal cartoon*, RIα CNB domains; *red cartoon with sticks*, RIα linker region containing the IS to PKAc). The serine residue of interest in RIα (Ser^101^, *red*) is depicted along with other critical residues at this binding interface, including 1) the pseudosubstrate alanine (Ala^99^) and *p* + 1 residue (Ile^100^) from the RIα linker region; 2) two residues from the αC-helix of PKAc (Gln^84^ and His^87^) that form direct hydrogen bonds with the hydroxyl group of serine 101; and 3) activation loop phosphorylation site (Thr(P)^197^) and its neighboring residues in PKAc (Arg^165^, Arg^189^, and Ser^195^). Hydrogen bonds are depicted as *dashed lines*.

cAMP-dependent protein kinase (PKAc; with α, β, and γ isoforms)^2^ is a pivotal cell signaling protein in eukaryotes (1–4). The catalytic activity of PKAc is in part controlled by four functionally non-redundant regulatory subunit proteins (RIα, RIβ, RIIα, and RIIβ) (5–8), which bind PKAc in tetrameric “holoenzyme” complexes to maintain kinase inactivity until stimulation with cAMP (1, 9). The structural assembly of PKA holoenzymes and allosteric activation mechanism triggered by cAMP binding to R-subunits is well-understood (9–15). However, unique biochemical features of RIα may serve to activate PKAc via non-canonical, cAMP-independent mechanisms. The linker–hinge region of PKA regulatory subunit proteins contains the autoinhibitory motif that allows for selective inhibition of PKAc at the kinase active site; however, this inhibitor sequence (IS) differs significantly between RI and RII (11, 16). Although RII subunits have a PKA consensus phosphorylation sequence (RR*X*S) that is phosphorylated by PKAc, RI subunits have a “pseudosubstrate” IS (RR*X*(A/G)) that is unable to be phosphorylated by PKAc; therefore, substrate competition is required to trigger full PKA holoenzyme dissociation (17). Additionally, RIα subunit has three serine residues in the linker region that are putative phosphorylation sites: Ser^77^, Ser^83^, and Ser^101^ (Fig. 1*A*) (18–20). Previously, the Ser^101^ residue (P + 2 to the pseudosubstrate IS) was shown to be an *in vitro* substrate of cGMP-dependent protein kinase (PKG) (21, 22). Given the nature of the *in vitro* methodology used previously to assess phosphorylation of this site, as well as the lack of identity of a well-defined PKG consensus phosphorylation sequence, the physiological relevance of this putative phosphorylation site remains uncharacterized. To expand upon this work, experiments were aimed at validating *in vitro* studies with purified recombinant proteins and determining whether this unique mechanism of PKA activation happens in cell culture models.

Recent structural information acquired in the last 10–15 years concerning the nature of binding between PKAc and RIα allows for some conjecture about how modification of Ser^101^ might lead to changes in type I PKA activity. The protein structure of the RIα–PKAc heterodimer was solved by X-ray crystallography techniques by Kim *et al.* (12), and this structure showed how the linker region of RIα binds the active site cleft of PKAc. The IS makes direct contacts with residues from both the N-lobe and C-lobe of PKAc, whereas the CNB-A domain binds distally to the C-lobe. Because of the pseudosubstrate nature of the IS in RIα, the high affinity binding of this motif to PKAc (in complex with ATP and two magnesium ions, Mg_2_ATP) presents a kinetic barrier for activation, whereas this is not critical for RII subunits capable of phosphorylation by PKAc (16, 23). The binding affinity of RIα and PKAc in the presence of Mg_2_ATP is 0.1 nm *versus* 200 nm in the absence of nucleotide (24). ATP also binds with an affinity of 60 nm in the RIα holoenzyme, whereas the *K_m_*/*K_d_* is 25 μm for the free protein. This gives a rationale for why modification of Ser^101^ could possibly perturb the binding interaction of the linker region of RIα to PKAc.

A close-up view of this interfacial region helps to illustrate the importance of this residue in maintaining proper binding interactions with PKAc (Fig. 1*B*). Upon analysis of polar interactions in this region, one can see that the hydroxyl moiety of Ser^101^ makes hydrogen bonds with residues from the αC-helix of PKAc (particularly residues Gln^84^ and His^87^). It is inferred that introduction of a phosphate group at this position will introduce steric hindrance that will likely alter the interaction of these residues. Furthermore, the interaction of Ser^101^ with Gln^84^ and His^87^ facilitates a hydrogen-bond network near the activation-loop phosphorylation site Thr^197^ in PKAc. Thus, we can further postulate that phosphorylation of Ser^101^ will also bring about a negative charge–charge repulsion effect caused by the juxtaposition of two phosphate groups within this binding interface. These effects in combination would lend to opening of the active site cleft of PKAc. Taking these observations under consideration, we hypothesized that phosphorylation of Ser^101^ by PKG would promote enhanced PKAc activity by removing the additional requirement of substrate competition in RIα holoenzyme activation. Finding evidence of direct PKG–PKA cross-talk in the context of cellular signaling would aid our understanding of how these two related pathways could regulate physiologically relevant systems where both proteins are expressed (*i.e.* smooth muscle and cardiac tissues) (25, 26).

## Results

### PKGIα phosphorylates RIα at serine 101 both in vitro and in cells

**Figure 2.**
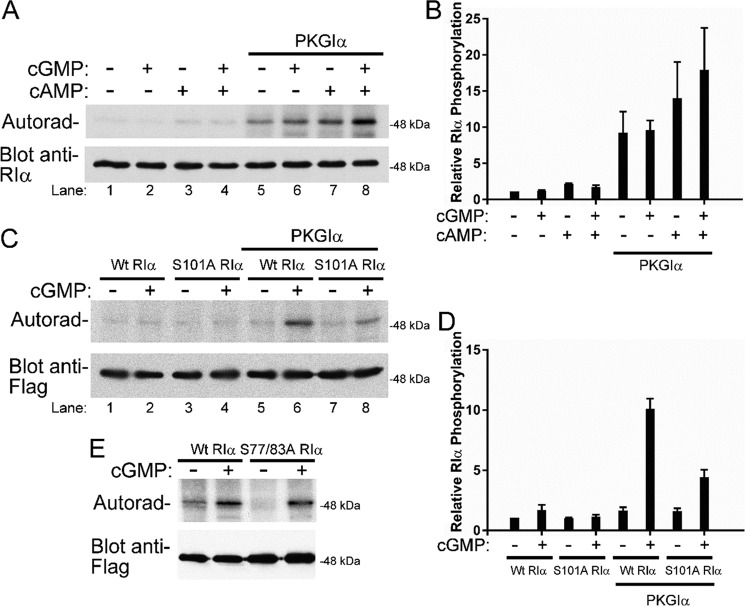
**PKGIα phosphorylates RIα *in vitro* and in cells.** *A*, the *upper panel* shows autoradiography of *in vitro* PKGΙα phosphorylation reactions conducted with purified recombinant bovine RIα in complex with PKAc (holoenzyme). For all reactions, 0.6 μg of holoenzyme was incubated with or without purified PKGIα in the presence of 1 μm of the indicated cyclic nucleotides. The *lower panel* shows equal loading via an anti-RIα immunoblot. *B*, densiometric quantification of three experiments performed as described in *A*, expressed as means ± S.D. Statistical significance is indicated by planned comparisons. *C*, autoradiography and immunoblot analysis of phosphorylation reactions performed in HEK293T cells overexpressing FLAG-tagged versions of either wildtype or S101A mutant RΙα protein, with and without the presence of PKGIα and/or 2 h of cGMP stimulus (*upper panel*). The *lower panel* shows equal expression and isolation of FLAG-tagged RIα proteins via an anti-FLAG immunoblot. *D*, densiometric quantification of three experiments performed as described in *C*, expressed as means ± S.D. Statistical significance is indicated by planned comparisons. *E*, the *upper panel* shows autoradiography of phosphorylation reactions performed in HEK293T cells overexpressing FLAG-tagged constructs of either WT or serine 77 and serine 83 mutant versions of RIα (S77/83A). All reactions were performed with overexpressed PKGIα with or without 2 h of 8-CPT-cGMP stimulus. The *lower panel* is an immunoblot using anti-FLAG antibodies as a loading control.

To extend previous reports demonstrating that PKA RIα could be phosphorylated by PKGIα, we performed *in vitro* phosphorylation reactions using purified recombinant bovine RIα (bRIα)–PKAc holoenzyme and PKGIα. The reactions contained [γ-^32^P]ATP, and phosphate incorporation was determined by autoradiography. We observed robust phosphorylation of bRIα only in the presence of PKGIα (Fig. 2*A*, compare *lanes 5–8* with *lanes 1–4*; quantification of data in Fig. 2*B*). RIα was phosphorylated by PKGIα to a similar extent in the absence and presence of cyclic nucleotides (*lanes 5–8*). These results are a bit unexpected, because cyclic nucleotide free and cAMP-bound PKGIα should not be as active as the cGMP-bound kinase. However, under the conditions used, it is likely that PKGIα activity was driven by PKGIα autophosphorylation and was cyclic nucleotide–independent. We originally reasoned that cAMP would disassociate bRIα from PKAc and thus provide easier access for PKGIα to phosphorylate Ser^101^; however, although there was a trend toward increased RIα phosphorylation in the presence of cAMP, the difference did not reach significance (compare *lane 5* with 7 and 6 with 8). Thus, it appears that holoenzyme association/dissociation has no effect on PKGIα's access to the phosphorylation site.

Next, we examined whether PKGIα could phosphorylate RIα in intact cells. HEK293T cells were transfected with expression vectors for PKGIα and FLAG-tagged WT or S101A-mutant human RΙα. The cells were labeled with [^32^P]orthophosphate, and the differences between WT and S101A-mutant RIα phosphorylation were compared in the presence or absence of PKGIα, as well as with and without 2-h stimulus with 8-CPT-cGMP (a membrane-permeable analog of cGMP). In cells transfected with WT RIα alone, we observed a small amount of basal RIα phosphorylation, which was slightly increased by treatment with 8-CPT-cGMP (Fig. 2*C*, compare *lanes 1* and *2*; quantification of data in Fig. 2*D*). In cells co-transfected with PKGIα, treatment with 8-CPT-cGMP induced a much higher level of RIα phosphorylation, which was dramatically reduced in cells transfected with PKGIα and S101A-mutant RIα (Fig. 2*C*, compare *lane 2* with *lane 6* and *lane 6* with *lane 8*). The *lower panel* of Fig. 2*C* is an immunoblot using anti-FLAG antibodies, showing an equal expression of transfected FLAG-tagged RIα in all samples tested. Our results indicate that RIα Ser^101^ is a specific phosphorylation target of PKGIα in cells.

In these cell-based experiments, the faint phosphorylation signal observed for RIα in cells expressing S101A-mutant RIα indicates the presence of other phosphorylation sites that are targeted in cells. To address this, two other putative phosphorylation sites within RIα protein, namely Ser^77^ and Ser^83^, were investigated (Fig. 2*E*). These two sites have been shown to be phosphorylated in human heart tissues in the context of ischemic heart disease (20) and thus may contribute to the background signal in our phosphorylation assays. Phosphorylation reactions were performed in HEK293T cells overexpressing FLAG-tagged constructs of either WT or a mutant version of RΙα protein with both Ser^77^ and Ser^83^ mutated to alanine. All reactions were conducted with overexpressed PKGIα either with or without 2 h of cGMP stimulus. In cells overexpressing WT protein, low levels of RIα phosphorylation in cells without cGMP stimulus were observed as seen in previous experiments. However, cells expressing the S77A/S83A double mutation of RIα did not yield phosphorylation signal without cGMP stimulus, indicating that all background signal observed in non-stimulated samples is due to extraneous in-cell phosphorylation of these two particular sites. As before, immunoblots using anti-FLAG antibodies were performed to show equal expression of RIα. These initial phosphorylation experiments performed under both *in vitro* and in-cell conditions have shown that serine 101 in RIα is indeed a *bona fide* phosphorylation target of PKGIα in mammalian cells.

### Mutation at serine 101 in RIα induces PKAc activity without cAMP stimulus in vitro and in cells

**Figure 3.**
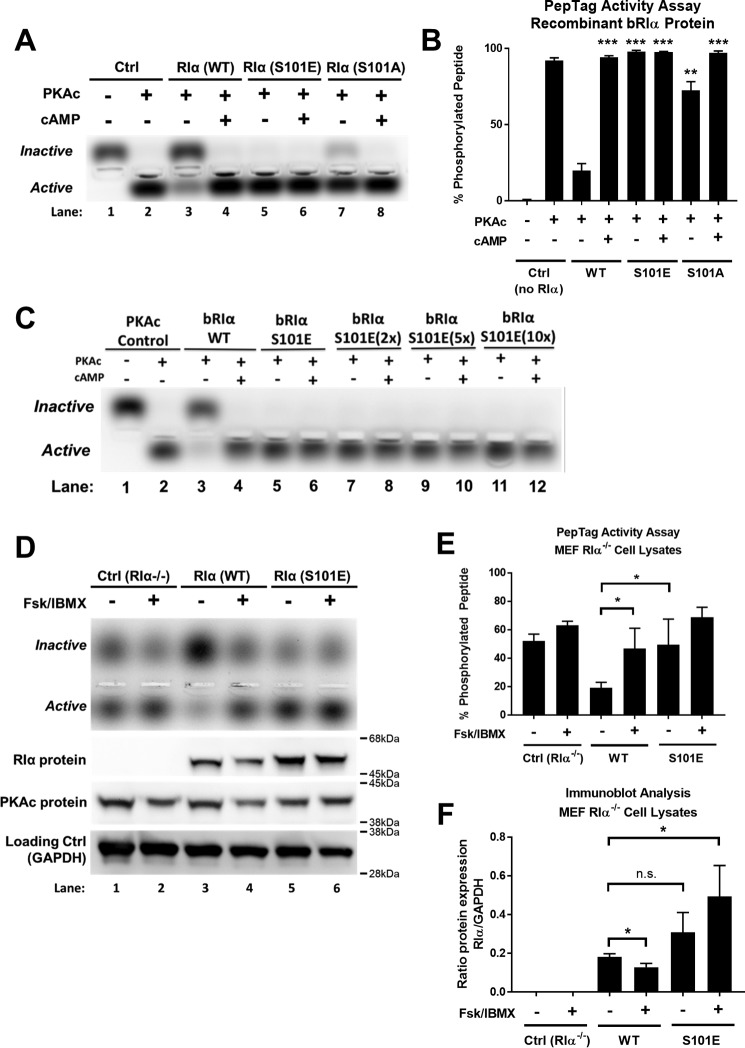
**Mutation of bovine RIα at serine 101 induces PKAc activity without cAMP stimulus *in vitro*.** *A*, analysis of *in vitro* PKAc kinase activity using the PepTag® activity assay kit, comparing the inhibitory capacity of either WT, phosphomimetic mutant (S101E), or alanine mutant (S101A) variants of purified bovine RIα protein (*n* = 3). *B*, densiometric quantification of three experiments performed as described in *A*, expressed as means ± S.D. Statistical significance is indicated as compared with RIα(WT) without cAMP. **, *p* < 0.001; ***, *p* < 0.0001. *C*, qualitative analysis of *in vitro* PKAc kinase activity using the PepTag® activity assay kit, comparing the inhibitory capacity of either WT or phosphomimetic mutant (S101E) variants of purified bovine RIα protein. In this experiment, full-length RIα S101E dimer protein was added in doses of progressively higher molar stoichiometry as compared with PKAc protein. *Lane 1*, no protein control; *lanes 2–12*, 200 nm PKAc; *lanes 3* and *4*, 120 nm WT dimer; *lanes 5* and *6*, 120 nm S101E dimer; *lanes 7* and *8*, 240 nm S101E dimer; *lanes 9* and *10*, 480 nm S101E dimer; *lanes 11* and *12*, 1.2 μm S101E dimer (*n* = 1). *D*, analysis of PKAc kinase activity using the PepTag® activity assay kit, comparing WT or phosphomimetic mutant (S101E) variants of human RIα overexpressed in MEF RIα^−/−^ cells (mouse embryonic fibroblasts with genetic knockout of *PRKAR1*α). To stimulate cAMP production in these cells, Fsk/IBMX (10 and 100 μm) were added for 10 min prior to cell lysis and subsequent activity assay (*n* = 3). The *lower panels* are immunoblots of RIα, PKAc, and glyceraldehyde-3-phosphate dehydrogenase (*GAPDH*) loading control for the given treatment conditions. *E*, densiometric quantification of three activity assay experiments performed as described in *D*, expressed as means ± S.D. Statistical significance is indicated as compared with WT without Fsk/IBMX. *, *p* < 0.05. *F*, densiometric quantification of three immunoblot experiments performed as described in *D*, expressed as means ± S.D. Statistical significance is indicated as compared with WT without Fsk/IBMX. *, *p* < 0.05. *Ctrl*, control.

As mentioned in the introduction, we hypothesized that phosphorylation of Ser^101^ would serve to increase PKAc activity in cells. Therefore, we developed mutations at this site in RIα, via site-directed mutagenesis of the serine to either a glutamate residue (S101E) or an alanine residue (S101A), to test whether this modification will weaken IS binding to the active site of PKAc and thus allow the competition of substrates to bind and be phosphorylated. After generating S101E and S101A mutant constructs for recombinant protein purification (full-length bovine RIα (1–379) in pRSET vector) and S101E mutant for mammalian cell transfection (full-length human RIα (1–379) in pcDNA3.1 vector), components of the PepTag® phosphorylation assay were used to assess differences in PKAc activation for wildtype *versus* mutant protein under both *in vitro* and in-cell conditions. Under *in vitro* conditions, WT RIα maintains PKAc in an inhibited state without cAMP stimulus; however, both S101E and S101A mutants display high PKAc activity under basal (cAMP-free) conditions (Fig. 3, *A* and *B*). Increasing S101E protein concentration from 2×, 5×, and 10× the relative stoichiometry of WT protein had no effect on inhibiting the observed high basal PKAc activity (Fig. 3*C*).

For in-cell analysis of PKAc activity, WT and S101E-mutant human RIα were overexpressed in MEF RIα^−/−^ cells (mouse embryonic fibroblasts with a genetic knockout of *PRKAR1*α). The RIα^−/−^ cell line allows for a null background to compare the addition of exogenous RIα overexpression. The relative degree of PKAc phosphorylation activity was compared between non-transfected cells (control RIα^−/−^) and cells overexpressing either WT or S101E-mutant human RIα (Fig. 3, *D* and *E*). Control cells displayed high substrate phosphorylation under non-stimulated conditions, thus serving as a positive control for PKAc activity and also confirming earlier observations that in the absence of RIα, PKA activity is not well-regulated even though other R-subunit isoforms are present. In close corroboration with *in vitro* data, overexpression of WT RIα in cells inhibited PKAc activity in the absence of Fsk/IBMX. In contrast to WT, cells expressing the S101E mutant showed high activity without cAMP stimulus, similarly as observed under *in vitro* conditions. We also conducted immunoblot analysis of RIα and PKAc expression in MEF RIα^−/−^ lysates for the corresponding activity assay samples examined (Fig. 3, *D* and *F*). Two major trends were observed: 1) in WT cells, the level of RIα was slightly decreased upon Fsk/IBMX stimulation; and 2) RIα expression appeared to be slightly higher in S101E cells as compared with cells expressing WT RIα (but only significant when comparing WT vehicle to S101E Fsk/IBMX samples). The competitive nature of the phosphorylation reactions employed suggests that the IS of RIα(S101E) must have a lowered affinity for the PKAc active site cleft; otherwise the PKA substrate would not be able to bind effectively to allow for phosphotransfer activity of PKAc.

Two explanations are possible for the aforementioned results. One possibility is that the RIα(S101E) mutant is simply unable to form a complex with PKAc; alternatively, the mutant RIα protein may still distally bind PKAc (via the R-subunit CNBs domains), even though the modified sequence in the IS region is loosely anchored to the PKAc active site cleft. Therefore, several techniques were explored to evaluate the relative capacity of S101E and S101A mutants of RIα to form holoenzyme.

### Ser^101^ mutant variants of RIα pulldown PKAc from HEK293T cell lysates via co-immunoprecipitation (co-IP)

**Figure 4.**
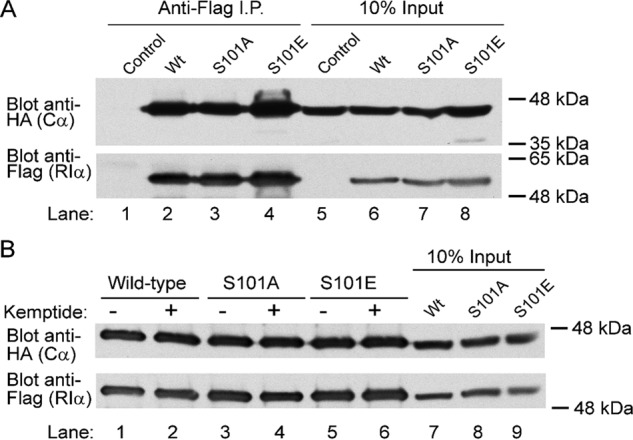
**Ser^101^ mutant variants of RIα pulldown PKAc from HEK293T cell lysates via co-IP.** Immunoblot analysis of co-IP reactions from HEK29T cell lysates, comparing cells overexpressing FLAG-tagged versions of either WT or serine 101 mutant variants (S101A and S101E) of RIα, was performed. The presence of HA-tagged PKAc protein (Cα) was examined in co-IP samples to qualitatively determine the state of RIα–PKAc complex formation in cells. In *A*, co-IP experiments were performed on untreated lysates, whereas in *B*, co-IP experiments were performed in the presence of 500 μm Kemptide substrate peptide. Both S101A and S101E mutants maintained binding to PKAc under all conditions tested, indicating that these mutations do not influence complex formation in cells. *I.P.*, immunoprecipitation.

To demonstrate that the Ser^101^ mutants of RIα can form holoenzyme complexes in cells, FLAG-tagged versions of human RIα were overexpressed in HEK29T cells, whereupon we assessed whether the S101A and S101E mutant variants of RIα were able to pull down overexpressed HA-tagged PKAc protein from cell lysates via co-immunoprecipitation. In this experiment, immunoblotting for the presence of HA-tagged protein in anti-FLAG IP samples can be used to qualitatively determine the state of holoenzyme complex formation in cells. We observed HA-tagged PKAc in the immunoprecipitate of all RIα proteins tested (WT, S101A, and S101E), signifying that these mutations do not influence the formation of heterodimer complexes between PKAc to RIα (Fig. 4*A*). Furthermore, co-immunoprecipitation of PKAc was also possible in the presence of excess substrate (Kemptide), indicating that excess substrate does not promote holoenzyme dissociation (Fig. 4*B*).

### Protein-fragment complementation assay (PCA) shows Ser^101^ mutants form cAMP-sensitive complexes in cells

**Figure 5.**
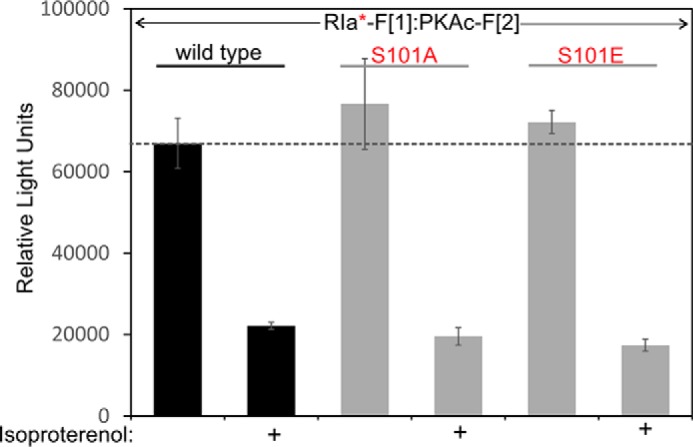
**PCA shows Ser^101^ mutants form cAMP-sensitive complexes in cells.** PCA (see “Experimental procedures”) was performed to measure RIα–PKAc protein association in isoproterenol-stimulated HEK293T cells, comparing WT to mutant RIα (S101A and S101E). In this assay, increased bioluminescence signal indicates complementation of the luciferase “split reporter” caused by protein–protein interaction mediated by holoenzyme association. Moreover, PKA holoenzyme dissociation is measured by luminescence signal (relative light units) decrease upon cellular stimulation with isoproterenol (a β-adrenergic receptor agonist known to stimulate cAMP production in cells). Shown is a representative of *n* = 3 independent experiments; ± standard deviation from triplicates.

To further confirm that mutant RIα forms stable holoenzyme complex in cells, a bioluminescent PCA (developed by Stefan and co-workers. (27); see “Experimental procedures”) was implemented. This assay uses differential tagging of PKA regulatory and catalytic subunit proteins with fragments of *Renilla* luciferase (RLuc) to create a “split-reporter” system that can be used to monitor R–C complex formation as a function of luciferase-fragment complementation. Upon cellular overexpression of RLuc-tagged versions of PKAc and either wildtype, S101A, or S101E variants of RIα, the relative degree of RIα–PKAc protein–protein interaction can be measured in intact cells. Moreover, PKA holoenzyme dissociation can be measured by luminescence signal decrease upon cellular stimulation with isoproterenol (a β-adrenergic receptor agonist known to stimulate cAMP production in cells). We observed that Ser^101^ mutants of RIα still displayed a similar degree of complex formation as compared with wildtype in non-stimulated cells (Fig. 5). Furthermore, bioluminescence signal was diminished upon addition of isoproterenol in all protein constructs tested, thus showing that the Ser^101^ mutant variants of RIα–PKAc complexes are still sensitive cAMP and thus allow for holoenzyme dissociation under stimulatory conditions.

### cAMP response of bRIα proteins shows high inhibitor binding for S101E, but not S101A mutant

**Figure 6.**
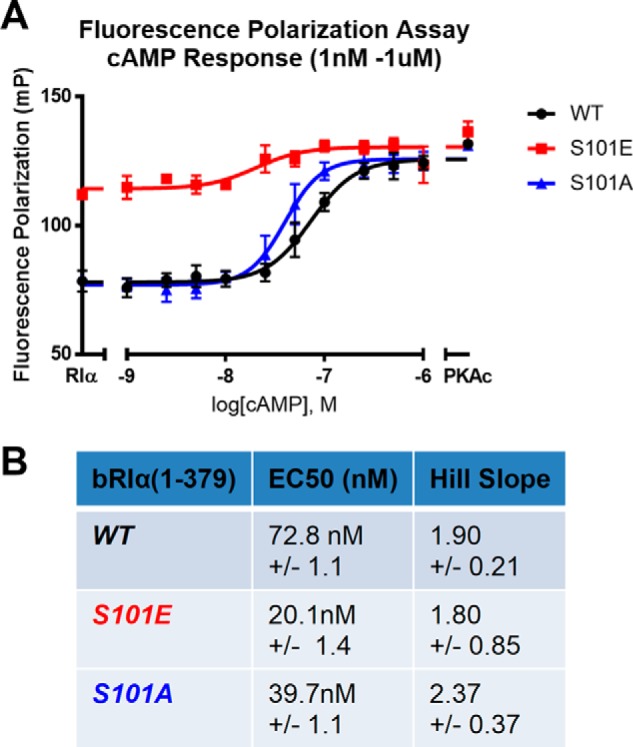
**cAMP response of bRIα proteins shows high inhibitor binding for S101E, but not S101A mutant.** A LiReC fluorescence polarization assay (see “Experimental procedures”) compared cAMP response between WT (*black*), S101E mutant (*red*), and S101A mutants (*blue*) forms of bRIα. *A*, graph of fluorescence polarization data reported in millipolarization units (*mP*). *B*, table of EC_50_ values and Hill slope. (Each condition was performed in quadruplicate, *n* = 4; *error bars* and table data are representative of ± S.E.).

For a more quantitative understanding of PKAc binding affinities for wildtype and mutant RIα subunits *in vitro*, we utilized the ligand-regulated competition (LiReC) fluorescence polarization assay (see “Experimental procedures”) (28). This competitive inhibitor-binding assay is used to detect the relative fluorescence polarization of FAM-IP20, a fluorescein-conjugated PKA inhibitor peptide that is derived from the heat stable protein kinase inhibitor. In the LiReC assay, the relative increase of the fluorescence polarization signal measured as the IP20 probe binds PKAc is indicative of the decrease in anisotropic tumbling of the immobilized inhibitor. First, a cAMP response assay was performed to compare FAM-IP20 binding to PKAc in the presence of either the WT, S101A, and S101E versions of RIα (Fig. 6). Whereas the S101A mutant behaved similarly as WT protein, the S101E mutant displayed significantly higher levels of FAM-IP20 binding in the absence of cAMP. In this instance, the S101E and S101A mutants showed lower EC_50_ values as compared with WT, yet Hill slope values for both mutants were not significantly changed.

### R-subunit inhibition response assay shows S101E binds PKAc with lower relative affinity

**Figure 7.**
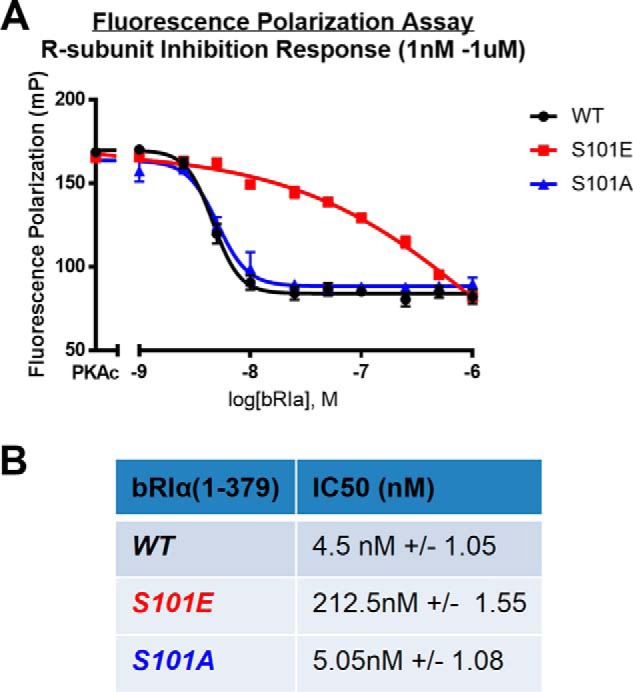
**R-subunit inhibition response assay shows that S101E binds PKAc with lower relative affinity.** LiReC fluorescence polarization assay (see “Experimental procedures”) compared inhibitory capacity of regulatory subunit protein between WT (*black*), S101E mutant (*red*), and S101A mutant (*blue*) forms of bRIα. *A*, graph of fluorescence polarization data reported in millipolarization units (*mP*). *B*, table of IC_50_ values and Hill slope. (Each condition was performed in quadruplicate, *n* = 4; *error bars* and table data are representative of ± S.E.).

To more conclusively determine whether RIα(S101E) with its modified linker region can functionally bind PKAc, the assay was modified to assess the inhibitory capacity for the RIα subunit in the presence of a constant concentration of PKAc (10 nm) and in the absence of cAMP (Fig. 7). WT, S101A, and S101E proteins were compared to discern any differences in formation of holoenzyme complexes for these serine 101 mutants. We observed that significantly higher levels of S101E mutant were required to out-compete FAM-IP20 for binding to PKAc (IC_50_^S101E^ = 212.5 nm ± 1.55). In contrast, S101A behaved very similar to WT RIα (IC_50_^WT^ = 4.5 nm ± 1.05; IC_50_^S101A^ = 5.05 nm ± 1.08), indicating that both proteins have an affinity for PKAc of less than 10 nm, which is the concentration of PKAc in the assay. These data suggest that the presence of the glutamic acid residue specifically and significantly affects the affinity of the RIα subunit to the PKAc active site, whereas the more conservative alanine substitution can function similar to unmodified wildtype protein at 10 nm concentrations. Thus, these experiments were able to illustrate that RIα(S101E) can functionally bind PKAc to out-compete FAM-IP20, but the relative inhibitory equilibrium constant was approximately 2 orders of magnitude higher compared with the wildtype RIα control.

## Discussion

**Figure 8.**
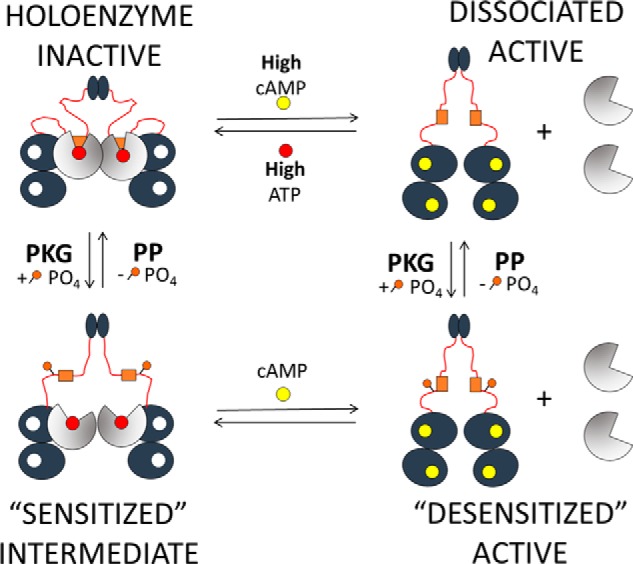
**PKG phosphorylation model of type I PKA activation.** A cartoon diagram of a modified, three-state model of type I PKA holoenzyme activation based on RIα phosphorylation by PKG is shown. Our model illustrates that type I holoenzyme may not transition directly from an inactive holoenzyme-complex state to a completely dissociated and active state. Instead, our data suggest that phosphorylation of serine 101 in RIα lowers the affinity between the IS of RIα and the active site of PKAc, thus leading to a “sensitized intermediate” state that displays PKAc catalytic activity while maintaining a holoenzyme complex configuration. This intermediate would then respond to physiological levels of cAMP toward a “desensitized” active state of the dissociated holoenzyme complex. Our model also denotes the potential for reversibility of linker region phosphorylation by protein phosphatases (*PP*).

To summarize the work presented here, we have revisited and additionally built upon a former concept within the field of protein kinase signaling, which highlights a potential cross-talk mechanism between the key contributors of cyclic nucleotide signaling in eukaryotic cells (PKA and PKG). Our evidence has validated that Ser^101^ in RIα is a target of PKG both *in vitro* and in cells, and furthermore we showed that introduction of a phosphorylation modification (or phosphomimetic mutation) at the serine 101 site in RIα leads to a state of heightened PKAc activity while still maintaining a RIα–PKAc complex. These data significantly shift the paradigm regarding our understanding of Type I PKA signaling, in that modification of the RIα linker region can serve to circumvent previously described kinetic restrictions of holoenzyme dissociation and thus trigger what we denote as “desensitized” PKAc activity. We have thus created a revised model of RIα holoenzyme activation to better illustrate how the effect of PKG phosphorylation could lead to significant changes in the equilibrium of RIα–PKAc association and dissociation (Fig. 8). In this model, modification of RIα leads a “sensitized intermediate” state of the RIα holoenzyme that is capable of phosphotransfer activity because of the lack of linker region accessibility at the PKAc active site. The addition of physiological levels of cAMP thus can trigger rapid dissociation of the holoenzyme complex, therefore creating a “desensitized” state of activity because of the reduced affinity of the modified RIα to reassociate with PKAc. Although this study focused upon PKG phosphorylation of Ser^101^ in RIα, our supplementary investigation of alternative phosphorylation sites in the linker region portion of the protein (*i.e.* Ser^77^ and Ser^83^) has further highlighted the potential significance of linker region modification as a regulatory mechanism for type I PKA in cells. Because these sites are differentially phosphorylated in heart tissues with and without onset of heart failure (20), it is possible that these linker region phosphorylation sites in RIα are specifically involved in regulating response to stress in the context of cardiac disease. Further investigation is also required to address the presumed role of protein phosphatases in allowing reversibility of phosphorylation at these linker region sites.

In terms of chemical equilibrium, we propose that modified RIα (*i.e.* the sensitized intermediate) has a significant (nearly 2 orders of magnitude) decrease in binding affinity to PKAc as compared with the 0.1 nm *K_d_* of the inactive holoenzyme. This assertion could very well explain discrepancies observed in related scientific literature, where it has been shown that elevation of cAMP under physiological conditions may not be sufficient to explain how PKA is activated upon stimulatory signaling in the cell (29). However, further examination is required to determine the exact nature of binding in the “sensitized intermediate” protein complex, with particular regard to 1) pseudosubstrate binding/accessibility to the active site cleft of PKAc, and 2) effects upon allosteric interactions in the modified holoenzyme. In future studies, surface plasmon resonance methods will be used to quantitatively determine the binding affinity of Ser^101^ mutants for PKAc. Utilization of alternative biophysical methodologies such as hydrogen/deuterium exchange mass spectrometry could also help to better explore the relative degree of conformational dynamics for WT and S101E proteins binding to PKAc by quantifying the degree of solvent exposure upon differing timescales of protein–protein interactions.

Although this study provides additional biochemical characterization of PKG phosphorylation of Ser^101^ in RIα in a cellular overexpression system, a final goal for this work is to assess the significance of this mechanism within *in vivo* systems. Because both PKG and PKA signaling play critical roles within cardiac and smooth muscle cell physiology (26, 30, 31), our ongoing research is aimed at detecting endogenous phosphorylation of RIα in differentiated muscle cell types, either treated with or without stimulus to drive PKG activity. Along this line of investigation, determining the role of oxidative stress signaling in the activation of this cross-talk mechanism will be of particular importance, because reactive nitrogen/oxygen signaling has been shown to result in alterations of heart physiology as manifested through both PKA and PKG (32–34). Given that both PKG and PKA have been implicated as targets in disease conditions such as dilated cardiomyopathy (35–37), heart failure with preserved ejection fractions (38, 39), and ischemia reperfusion injury (40, 41), characterization of RIα phosphorylation within endogenous tissues may further bolster the significance of this mechanism within the context of clinically relevant cardiovascular diseases.

## Experimental procedures

### Materials

Antibodies for PKAc and RIa were from BD Biosciences (San Jose, CA); antibodies for glyceraldehyde-3-phosphate dehydrogenase were from Santa Cruz Biotechnology (Dallas, TX). Anti-HA and anti-species secondary antibodies were obtained from Santa Cruz Biotechnology. Other antibodies, chemicals, and reagents were obtained from Sigma unless otherwise stated.

### Recombinant DNA and site-directed mutagenesis

Constructs of full-length human RIa gene (hRIa 1–379) incorporated into the pCDNA3.3 mammalian expression vector were used for cellular transfection experiments in MEFs. Point mutations (hRIa[S101A] and hRIa[S101E]) were introduced by site-directed mutagenesis via QuikChange® PCR as per the manufacturer's instructions (Stratagene, La Jolla, CA). The same genetic material was also incorporated into pRSET-Xa3 vector for bacterial recombinant protein expression. Point mutations (bRIa[S101A] and bRIa[S101E]) were introduced into both constructs by site-directed mutagenesis via QuikChange® PCR as per the manufacturer's instructions (Stratagene). FLAG-tagged RIα was constructed by PCR using an untagged RIα vector as a template. The PCR product was digested and inserted into the pFLAG-D expression vector. The S101A and S101E mutant RIα constructs were generated using overlapping extension PCR. All constructs that went through a PCR step were sequenced to ensure that the expected coding sequence was present.

### Expression and purification of recombinant proteins from Escherichia coli

The C-subunit was expressed in *E. coli* and purified as described (42). Full-length (1–379) constructs of either wildtype, S101E, and S101A mutants of bRIa (in pRSET-Xa3 vector) were expressed in BL21(DE3) cells and purified by cAMP affinity chromatography in 20 mm MES (pH 6.5), 100 mm NaCl, 2 mm EGTA, 2 mm EDTA, and 5 mm DTT. RIα proteins were further purified on a gel filtration column using Superdex 200 and concentrated for purposes of biochemical assays using Amicon® centrifugal filters (EMD Millipore, Billerica, MA).

### In vitro PKA RIα phosphorylation by PKGIα

To examine RIα–PKAc holoenzyme phosphorylation by PKGIα, purified PKGIα (43) was added to a 20-μl reaction mix containing 0.6 μg of wildtype RIα–PKAc holoenzyme complex, 30 mm HEPES (pH 7.4), 10 mm MgCl_2_, 10 mm β-glycerol phosphate, 100 mm ATP, 0.05 mCi of [γ-^32^P]ATP, and 1 μm of the indicated cyclic nucleotides. The reactions were incubated at 30 °C for 2 h. The reactions were terminated by adding 20 μl of 2× SDS-PAGE loading buffer containing 100 mm EDTA followed by boiling. Phosphate incorporation was determined by SDS-PAGE/autoradiography. Densiometric analysis was performed using ImageJ.

### Cell culture: Growth, transfection, and treatments

HEK293T and mouse embryonic fibroblast (MEF) cells were used throughout this study. Propagating cultures were grown in 10-cm dishes using Dulbecco's modified Eagle's medium supplemented with 10% fetal bovine serum. MEF cultures contained 1% penicillin/streptomycin, unless otherwise stated.

### Phosphorylation of RIα in HEK293T cells

HEK293T cells were grown in Dulbecco's modified Eagle's medium with 10% fetal bovine serum in a 37 °C incubator with a 5% CO_2_ atmosphere. The day before transfection, the cells were spit into a 6-well cluster dish such that the cells were 90–95% confluent at the time of transfection. The cells were transfected with the indicated expression constructs with Lipofectamine 2000 using conditions recommended by the manufacturer (Thermo Fisher). The next day, the medium was removed, and 1 ml of phosphate-free media and 100 μCi of ^32^PO_4_ was added to each well. The cells were incubated at 37 °C for 2 h, and then 250 μm 8-CPT-cGMP was added to the appropriate wells. The cells were incubated for an additional 2 h at 37 °C, at which point the medium was removed, the cells were washed once with ice-cold PBS, and the cells were lysed on the plate in PBS, 0.1% Nonidet P-40, 1× protease inhibitor mixture (Calbiochem, San Diego, CA), and 1× HALT phosphatase inhibitor (Cell Signaling Technology, Danvers, MA). Cleared lysates were added to 20 μl of anti-FLAG affinity gel and incubated for 1 h at 4 °C with constant mixing. The beads were washed and boiled in 30 μl of 1× SDS sample buffer, and phosphorylation was analyzed by SDS-PAGE/autoradiography. The relative amount of immunoprecipitated FLAG-tagged RIα was determined by immunoblotting.

### PepTag® non-radioactive PKA phosphorylation assay

Assays using both cell lysates and recombinant proteins were performed in a modified procedure from the manufacturer's directions. For cell-based experiments, MEF cell samples were plated on 6-well dishes at passage 3. Transfections were conducted using Lipofectamine 2000® (Thermo Fisher) as per the manufacturer's directions. For activity experiments, MEF cells were serum-starved for 2 h before conducting 10 min of Fsk/IBMX stress treatment (*i.e.* 20 μm forskolin and 100 μm IBMX). 10 μl of cell lysate (of normalized protein concentration) was added to 5 μl each of “5× peptide,” “5× buffer,” and distilled water for all reactions. The samples were mixed and incubated at room temperature for 30 min and then heat-inactivated at 95 °C for 10 min. For recombinant protein experiments*:* RIα and PKAc proteins were incubated *in situ* at a 1.2:1 molar stoichiometry within a 10-μl volume (for RIα(1–379) dimer protein, 5 μl of 1.2 μm R-subunit was added to 5 μl of 2 μm PKAc). Then 5 μl each of 5× peptide, 5× buffer, and either 5 mm cAMP or distilled water was added to start the reactions. Samples were mixed and incubated at room temperature for 30 min and then heat-inactivated at 95 °C for 10 min. For all experiments, phosphopeptides were separated by 0.8% agarose gel as per the manufacturer's directions. Densiometric analysis of gel images was performed using ImageJ. Activity is expressed as the percentage of phosphorylated peptide, calculated by the ratio of phosphorylated peptide signal divided by total signal.

### Immunoblotting

SDS-PAGE samples were prepared using 5× Laemmli buffer (100 mm Tris-HCl, pH 7.0, 10% SDS, 50% glycerol, 0.01% bromphenol blue). Protein concentrations were normalized between samples, and then SDS-PAGE was performed using NuPAGE 4–12% gradient gels (either 10-, 12-, or 15-well) in MES running buffer (55 mm MES, 10 mm Tris-base, 1 mm EDTA, 0.1% SDS). The gels were resolved at 150 V for 70 min and then subsequently transferred to 0.45-μm nitrocellulose membranes (200 mA/blot for 60 min). The membranes were blocked using 5% milk protein (or with 5% BSA for phosphoblots) in 1× PBS, 0.05% Tween for 60 min at room temperature, and then primary antibodies were incubated overnight at 4 °C in 1× PBS, 0.05% Tween 20 or 1× TBS, 0.1% Tween 20 at the concentrations specified by the manufacturer. Densiometric analysis was performed using ImageJ.

### Co-immunoprecipitation

Expression vectors encoding HA-tagged PKAc and WT or Ser^101^ mutant FLAG-tagged RIα, or empty vector were transfected into 293T cells using Lipofectamine 2000. After 20 h, the cells were lysed in PBS, 0.1% Nonidet P-40 with 1× protease inhibitor mixture. The lysates were cleared by centrifugation at 16,000 × *g* for 10 min, and supernatants were incubated with anti-FLAG M2 affinity gel for 1 h at 4 °C with constant mixing. The beads were washed three times with PBS, 0.1% Nonidet P-40, proteins were eluted by boiling in 2× Laemmli buffer, and bound proteins were analyzed by SDS-polyacrylamide gel electrophoresis followed by immunoblotting. For some experiments, 500 μm Kemptide was added during the immunoprecipitations.

### PCA using fragmented RLuc

Plasmid constructs of PKAc tagged with F1 fragment (PKAc-F1) and RIα tagged with F2 fragment (RIα-F2) were generated previously (27). S101E and S101A mutations of RIα were introduced into RIα-F2 by site-directed mutagenesis via QuikChange® PCR as per the manufacturer's instructions (Stratagene). PKAc-F1 and either WT, S101E, or S101A versions of RIα-F2 plasmids were co-transfected into HEK293T cells, and bioluminescence signal was compared under basal conditions, as well as with 15 min of 10 nm isoproterenol stimulus.

### LiReC fluorescence polarization assay

Use of the LiReC assay has been described previously (28). All reactions were conducted in assay buffer (50 mm MOPS, pH 7.0, 35 mm NaCl, 10 mm MgCl_2_, 1 mm ATP, 2 mm DTT, and 0.005% Triton X-100). End-point fluorescence polarization measurements were performed using a GENios Pro® plate-reader spectrometer (Tecan, Mannendorf, Switzerland), configured with 485-nm excitation and 535-nm emission filters and using optimal gain settings. Concentrations of proteins, [5/6-FAM]-IP20, and cAMP for each experimental method are listed below: for cAMP response experiments, 12.0 nm PKA catalytic subunit, 14.4 nm PKA regulatory subunit bRIα (91–379) or bRIα (1-379) (based on monomer concentration), 2.0 nm [5/6-FAM]-IP20, 1 nm-1 μm cAMP (dissolved in 1× buffer without ATP or DTT); and for R-subunit inhibition response experiments, 10.0 nm PKA catalytic subunit, 1 nm-1 μm PKA regulatory subunit bRIα(91–379) or bRIα(1–379) (based on monomer concentration), 1.67 nm [5/6-FAM]-IP20.

### Statistics

All activity, immunoblot, and PCA data are presented as means ± S.D., and all fluorescence polarization assay data are presented as means ± S.E. GraphPad Prism 4 software (GraphPad Software, Inc., San Diego, CA) was used for all statistical analysis. Statistical analyses were performed by unpaired Student's *t* test of planned comparisons.

## Author contributions

K. J. H. designed and conducted most of the experiments, built expression constructs, generated recombinant proteins, analyzed the results, and wrote most of the paper. D. E. C. built expression constructs, conducted radioactive phosphorylation and co-immunoprecipitation experiments, and analyzed results. A. R. conducted protein complementation assay experiments and analyzed results. E. S. conducted protein complementation assay experiments, analyzed results, and provided materials for the study. H. H. P. provided material and facilities for the study and wrote the paper with K. J. H. S. S. T. conceived the idea for the project, provided material and facilities for the study, and wrote the paper with K. J. H.

## Footnotes

2 The abbreviations used are:

PKAc: protein kinase A catalytic subunit
PKG: protein kinase G
IS: inhibitor sequence
bRIα: recombinant bovine PKA regulatory subunit RIα
MEF: mouse embryonic fibroblast
PCA: protein-fragment complementation assay
RLuc: *Renilla* luciferase
LiReC: ligand-regulated competition
HA: hemagglutinin
CNB: cyclic nucleotide binding
Fsk: forskolin
IBMX: isobutylmethylxanthine
co-IP: co-immunoprecipitation.

## References

[1] Taylor S. S., Buechler J. A., and Yonemoto W. (1990) cAMP-dependent protein kinase: framework for a diverse family of regulatory enzymes. Annu. Rev. Biochem. 59, 971–1005 10.1146/annurev.bi.59.070190.004543 2165385

[2] Uhler M. D., Carmichael D. F., Lee D. C., Chrivia J. C., Krebs E. G., and McKnight G. S. (1986) Isolation of cDNA clones coding for the catalytic subunit of mouse cAMP-dependent protein kinase. Proc. Natl. Acad. Sci. U.S.A. 83, 1300–1304 10.1073/pnas.83.5.1300 3456589 PMC323063

[3] Uhler M. D., Chrivia J. C., and McKnight G. S. (1986) Evidence for a second isoform of the catalytic subunit of cAMP-dependent protein kinase. J. Biol. Chem. 261, 15360–15363 3023318

[4] Beebe S. J., Øyen O., Sandberg M., Frøysa A., Hansson V., and Jahnsen T. (1990) Molecular cloning of a tissue-specific protein kinase (Cγ) from human testis: representing a third isoform for the catalytic subunit of cAMP-dependent protein kinase. Mol. Endocrinol. 4, 465–475 10.1210/mend-4-3-465 2342480

[5] Lee D. C., Carmichael D. F., Krebs E. G., and McKnight G. S. (1983) Isolation of a cDNA clone for the type I regulatory subunit of bovine cAMP-dependent protein kinase. Proc. Natl. Acad. Sci. U.S.A. 80, 3608–3612 10.1073/pnas.80.12.3608 6190178 PMC394099

[6] Clegg C. H., Cadd G. G., and McKnight G. S. (1988) Genetic characterization of a brain-specific form of the type I regulatory subunit of cAMP-dependent protein kinase. Proc. Natl. Acad. Sci. U.S.A. 85, 3703–3707 10.1073/pnas.85.11.3703 3375237 PMC280286

[7] Scott J. D., Glaccum M. B., Zoller M. J., Uhler M. D., Helfman D. M., McKnight G. S., and Krebs E. G. (1987) The molecular cloning of a type II regulatory subunit of the cAMP-dependent protein kinase from rat skeletal muscle and mouse brain. Proc. Natl. Acad. Sci. U.S.A. 84, 5192–5196 10.1073/pnas.84.15.5192 3037538 PMC298820

[8] Jahnsen T., Hedin L., Kidd V. J., Beattie W. G., Lohmann S. M., Walter U., Durica J., Schulz T. Z., Schiltz E., and Browner M. (1986) Molecular cloning, cDNA structure, and regulation of the regulatory subunit of type II cAMP-dependent protein kinase from rat ovarian granulosa cells. J. Biol. Chem. 261, 12352–12361 2427518

[9] Taylor S. S., Ilouz R., Zhang P., and Kornev A. P. (2012) Assembly of allosteric macromolecular switches: lessons from PKA. Nat. Rev. Mol. Cell Biol. 13, 646–658 10.1038/nrm3432 22992589 PMC3985763

[10] Shabb J. B., and Corbin J. D. (1992) Cyclic nucleotide-binding domains in proteins having diverse functions. J. Biol. Chem. 267, 5723–5726 1313416

[11] Taylor S. S., Kim C., Vigil D., Haste N. M., Yang J., Wu J., and Anand G. S. (2005) Dynamics of signaling by PKA. Biochim. Biophys. Acta 1754, 25–37 10.1016/j.bbapap.2005.08.024 16214430

[12] Kim C., Cheng C. Y., Saldanha S. A., and Taylor S. S. (2007) PKA-I holoenzyme structure reveals a mechanism for cAMP-dependent activation. Cell 130, 1032–1043 10.1016/j.cell.2007.07.018 17889648

[13] Wu J., Brown S. H., von Daake S., and Taylor S. S. (2007) PKA type IIα holoenzyme reveals a combinatorial strategy for isoform diversity. Science 318, 274–279 10.1126/science.1146447 17932298 PMC4036697

[14] Zhang P., Smith-Nguyen E. V., Keshwani M. M., Deal M. S., Kornev A. P., and Taylor S. S. (2012) Structure and allostery of the PKA RIIβ tetrameric holoenzyme. Science 335, 712–716 10.1126/science.1213979 22323819 PMC3985767

[15] Ilouz R., Bubis J., Wu J., Yim Y. Y., Deal M. S., Kornev A. P., Ma Y., Blumenthal D. K., and Taylor S. S. (2012) Localization and quaternary structure of the PKA RIβ holoenzyme. Proc. Natl. Acad. Sci. U.S.A. 109, 12443–12448 10.1073/pnas.1209538109 22797896 PMC3411989

[16] Vigil D., Blumenthal D. K., Heller W. T., Brown S., Canaves J. M., Taylor S. S., and Trewhella J. (2004) Conformational differences among solution structures of the type Iα, IIα and IIβ protein kinase A regulatory subunit homodimers: role of the linker regions. J. Mol. Biol. 337, 1183–1194 10.1016/j.jmb.2004.02.028 15046986

[17] Døskeland S. O., Maronde E., and Gjertsen B. T. (1993) The genetic subtypes of cAMP-dependent protein kinase: functionally different or redundant? Biochim. Biophys. Acta 1178, 249–258 10.1016/0167-4889(93)90201-Y 8395890

[18] Boeshans K. M., Resing K. A., Hunt J. B., Ahn N. G., and Shabb J. B. (1999) Structural characterization of the membrane-associated regulatory subunit of type I cAMP-dependent protein kinase by mass spectrometry: identification of Ser81 as the *in vivo* phosphorylation site of RIα. Protein Sci. 8, 1515–1522 10.1110/ps.8.7.1515 10422841 PMC2144381

[19] Gupte R. S., Traganos F., Darzynkiewicz Z., and Lee M. Y. (2006) Phosphorylation of RIα by cyclin-dependent kinase CDK 2/cyclin E modulates the dissociation of the RIα-RFC40 complex. Cell Cycle 5, 654–661 10.4161/cc.5.6.2576 16582606

[20] Han Y. S., Arroyo J., and Ogut O. (2013) Human heart failure is accompanied by altered protein kinase A subunit expression and post-translational state. Arch. Biochem. Biophys. 538, 25–33 10.1016/j.abb.2013.08.002 23942052 PMC3879134

[21] Geahlen R. L., Allen S. M., and Krebs E. G. (1981) Effect of phosphorylation on the regulatory subunit of the type I cAMP-dependent protein kinase. J. Biol. Chem. 256, 4536–4540 6260803

[22] Geahlen R. L., Carmichael D. F., Hashimoto E., and Krebs E. G. (1982) Phosphorylation of cAMP-dependent protein kinase subunits. Adv. Enzyme Reg. 20, 195–209 10.1016/0065-2571(82)90016-4 6287816

[23] Martin B. R., Deerinck T. J., Ellisman M. H., Taylor S. S., and Tsien R. Y. (2007) Isoform-specific PKA dynamics revealed by dye-triggered aggregation and DAKAP1α-mediated localization in living cells. Chem. Biol. 14, 1031–1042 10.1016/j.chembiol.2007.07.017 17884635

[24] Herberg F. W., and Taylor S. S. (1993) Physiological inhibitors of the catalytic subunit of cAMP-dependent protein kinase: effect of magnesium-ATP on protein–protein interactions. Biochemistry 32, 14015–14022 10.1021/bi00213a035 8268180

[25] Boerth N. J., Dey N. B., Cornwell T. L., and Lincoln T. M. (1997) Cyclic GMP-dependent protein kinase regulates vascular smooth muscle cell phenotype. J. Vasc. Res. 34, 245–259 10.1159/000159231 9256084

[26] Lincoln T. M., Dey N., and Sellak H. (2001) Invited review: cGMP-dependent protein kinase signaling mechanisms in smooth muscle: from the regulation of tone to gene expression. J. Appl. Physiol. 91, 1421–1430 10.1152/jappl.2001.91.3.1421 11509544

[27] Röck R., Bachmann V., Bhang H. E., Malleshaiah M., Raffeiner P., Mayrhofer J. E., Tschaikner P. M., Bister K., Aanstad P., Pomper M. G., Michnick S. W., and Stefan E. (2015) *In-vivo* detection of binary PKA network interactions upon activation of endogenous GPCRs. Sci. Rep. 5, 11133 10.1038/srep11133 26099953 PMC4477410

[28] Saldanha S. A., Kaler G., Cottam H. B., Abagyan R., and Taylor S. S. (2006) Assay principle for modulators of protein–protein interactions and its application to non-ATP-competitive ligands targeting protein kinase A. Anal. Chem. 78, 8265–8272 10.1021/ac061104g 17165815 PMC3435108

[29] Boras B. W., Kornev A., Taylor S. S., and McCulloch A. D. (2014) Using Markov state models to develop a mechanistic understanding of protein kinase A regulatory subunit RIα activation in response to cAMP binding. J. Biol. Chem. 289, 30040–30051 10.1074/jbc.M114.568907 25202018 PMC4208011

[30] Keely S. L., and Corbin J. D. (1977) Involvement of cAMP-dependent protein kinase in the regulation of heart contractile force. Am. J. Physiol. 233, H269–H275 196511 10.1152/ajpheart.1977.233.2.H269

[31] Walter U. (1989) Physiological role of cGMP and cGMP-dependent protein kinase in the cardiovascular system. Reviews of Physiology, Biochemistry and Pharmacology, Vol. 113, pp. 41–88, Springer, Berlin Heidelberg 10.1007/BFb0032675 2560585

[32] Brennan J. P., Bardswell S. C., Burgoyne J. R., Fuller W., Schröder E., Wait R., Begum S., Kentish J. C., and Eaton P. (2006) Oxidant-induced activation of type I protein kinase A is mediated by RI subunit interprotein disulfide bond formation. J. Biol. Chem. 281, 21827–21836 10.1074/jbc.M603952200 16754666

[33] Burgoyne J. R., and Eaton P. (2009) Transnitrosylating nitric oxide species directly activate type I protein kinase A, providing a novel adenylate cyclase-independent cross-talk to β-adrenergic-like signaling. J. Biol. Chem. 284, 29260–29268 10.1074/jbc.M109.046722 19726669 PMC2785556

[34] Burgoyne J. R., and Eaton P. (2010) Oxidant sensing by protein kinases A and G enables integration of cell redox state with phosphoregulation. Sensors 10, 2731–2751 10.3390/s100402731 22319269 PMC3274199

[35] Antos C. L., Frey N., Marx S. O., Reiken S., Gaburjakova M., Richardson J. A., Marks A. R., and Olson E. N. (2001) Dilated cardiomyopathy and sudden death resulting from constitutive activation of protein kinase A. Circ. Res. 89, 997–1004 10.1161/hh2301.100003 11717156

[36] Aye T.-T., Soni S., van Veen T. A., van der Heyden M. A., Cappadona S., Varro A., de Weger R. A., de Jonge N., Vos M. A., Heck A. J., and Scholten A. (2012) Reorganized PKA-AKAP associations in the failing human heart. J. Mol. Cell. Cardiol. 52, 511–518 10.1016/j.yjmcc.2011.06.003 21712045

[37] Frantz S., Klaiber M., Baba H. A., Oberwinkler H., Völker K., Gassner B., Bayer B., Abesser M., Schuh K., Feil R., Hofmann F., and Kuhn M. (2013) Stress-dependent dilated cardiomyopathy in mice with cardiomyocyte-restricted inactivation of cyclic GMP-dependent protein kinase I. Eur. Heart J. 34, 1233–1244 10.1093/eurheartj/ehr445 22199120 PMC3631523

[38] van Heerebeek L., Hamdani N., Falcão-Pires I., Leite-Moreira A. F., Begieneman M. P., Bronzwaer J. G., van der Velden J., Stienen G. J., Laarman G. J., Somsen A., Verheugt F. W., Niessen H. W., and Paulus W. J. (2012) Low myocardial protein kinase G activity in heart failure with preserved ejection fraction. Circulation 126, 830–839 10.1161/CIRCULATIONAHA.111.076075 22806632

[39] Tschöpe C., Van Linthout S., Spillmann F., Klein O., Biewener S., Remppis A., Gutterman D., Linke W. A., Pieske B., Hamdani N., and Roser M. (2016) Cardiac contractility modulation signals improve exercise intolerance and maladaptive regulation of cardiac key proteins for systolic and diastolic function in HFpEF. Int. J. Cardiol. 203, 1061–1066 10.1016/j.ijcard.2015.10.208 26638055

[40] Murphy E., and Steenbergen C. (2008) Mechanisms underlying acute protection from cardiac ischemia-reperfusion injury. Physiol. Rev. 88, 581–609 10.1152/physrev.00024.2007 18391174 PMC3199571

[41] Schulz R., Kelm M., and Heusch G. (2004) Nitric oxide in myocardial ischemia/reperfusion injury. Cardiovasc. Res. 61, 402–413 10.1016/j.cardiores.2003.09.019 14962472

[42] Sutherland E. W., and Rall T. W. (1958) Fractionation and characterization of a cyclic adenine ribonucleotide formed by tissue particles. J. Biol. Chem. 232, 1077–1091 13549488

[43] Kalyanaraman H., Zhuang S., Pilz R. B., and Casteel D. E. (2017) The activity of cGMP-dependent protein kinase Iα is not directly regulated by oxidation-induced disulfide formation at cysteine 43. J. Biol. Chem. 292, 8262–8268 10.1074/jbc.C117.787358 28360102 PMC5437233

